# Diamine Crosslinked Addition-Type Diblock Poly(Norbornene)s-Based Anion Exchange Membranes with High Conductivity and Stability for Fuel Cell Applications

**DOI:** 10.3390/polym16243534

**Published:** 2024-12-18

**Authors:** Quan Li, Xiaohui He, Ling Feng, Jia Ye, Wenjun Zhang, Longming Huang, Defu Chen

**Affiliations:** 1 School of Physics and Materials Science, Nanchang University, 999 Xuefu Avenue, Nanchang 330031, Chinalynnfengling@email.ncu.edu.cn (L.F.); yj18879968369@163.com (J.Y.); 15079439505@163.com (L.H.); 2 School of Civil Engineering and Architecture, Nanchang University, 999 Xuefu Avenue, Nanchang 330031, China

**Keywords:** anion exchange membranes, hydrophilic crosslinking, flexible alkoxy spacer, conductivity, alkaline stability

## Abstract

Anion exchange membranes (AEMs) as a kind of important functional material are widely used in fuel cells. However, synthetic AEMs generally suffer from low conductivity, poor alkaline stability, and poor dimensional stability. Constructing efficient ion transport channels is widely regarded as one of the most effective strategies for developing AEMs with high conductivity and low swelling ratio. Herein we demonstrate a versatile strategy to prepare the AEMs with both high conductivity and excellent alkali stability via all-carbon hydrogen block copolymer backbone hydrophilic crosslinking and introducing flexible alkoxy spacer chains. Additionally, we investigated the impact of the crosslinking degree on the AEMs’ performances. It was found that the dosage of the hydrophilic crosslinker has a significant impact on the construction of efficient ion transport channels in the AEMs. Amazingly, the CL30-aPNB-TMHDA-TMA exhibited the highest hydroxide conductivity (138.84 mS cm^−1^), reasonable water uptake (54.96%), and a low swelling ratio (14.07%) at 80 °C. Meanwhile, the membrane showed an excellent alkaline stability in a 1 M NaOH solution at 80 °C for 1008 h (ion exchange capacity (IEC) and OH^−^ conductivity remained at 91.9% and 89.12%, respectively). The single cells assembled with CL30-aPNB-TMHDA-TMA exhibited a peak power density of 266.2 mW cm^−2^ under a current density of 608 mA cm^−2^ at 80 °C. The novel developed composite strategy of flexible alkoxy side chains with hydrophilic crosslinking modification is potentially promised to be an effective approach to develop the high-performance AEMs.

## 1. Introduction

Anion exchange membrane fuel cells (AEMFCs) have attracted widespread attention in the past decades due to their incomparable merits, such as the faster kinetics of oxygen reduction reactions (ORRs), non-noble metal catalysts, and the low fuel permeation [1,2,3,4,5]. As one of the core components of AEMFCs, anion exchange membranes (AEMs) possess the crucial function of transporting anions and separating the fuel. An ideal AEM should have high anion conductivity, appropriate dimensional stability, and excellent alkaline stability under high pH conditions [6,7,8]. However, the currently developed AEMs are unable to fully satisfy the growing requirements in these fields. Specially, these AEMs often suffer from insufficient alkaline stability and the pernicious “trade-off” between ion conductivity and dimensional stability [9,10,11,12]. Therefore, much research has been devoted to improving the performance of AEMs by molecular design.

AEMs are composed of polymer backbones and cationic groups through a certain connection [13,14]. At present, various polymer materials have been used to prepare AEMs, including polyolefin [15,16], polyphenylene ether [17,18], polystyrene [19,20], polysulfone [21,22], polyaryl ether ketone [23,24], polybenzimidazole (PBI) [25,26], and polynorbornene [27,28,29,30], etc. For polymer systems, polynorbornene has demonstrated excellent chemical stability, good mechanical properties, and good thermal stability due to its unique cyclic structure, attracting extensive attention in recent years [31,32]. Chen et al. [33] prepared a series of ring opening metathesis-type poly(norbornene)s AEMs. The results indicated that after immersion in 1 M NaOH at 80 °C for 792 h, the hydroxide conductivity remained almost unchanged, maintaining its initial value. He et al. [34] prepared addition-type polynorbornene-based AEMs, CL-aP(NB-O-Br-*b*-NB-MHE), and because of the fully hydrogenated bicyclopentane structure, all these AEMs not only showed excellent alkaline stability, but also exhibited sufficient dimensional, thermal, and mechanical stability. Therefore, polynorbornene is an exceptional polymeric material for the preparation of high-performance AEMs.

In addition to polymer backbones, the application of different cationic groups in fuel cells has been studied, such as quaternary ammoniums [35,36,37], imidazolium [38,39], guanidinium [40,41], and organic-metal cations [42,43]. For cationic groups, quaternary ammoniums have been extensively studied due to their excellent dissociation ability and hydroxide conductivity. However, all cationic groups, including quaternary ammonium, inevitably degrade in harsh alkaline environments. Especially, benzyltrimethylammonium is susceptible to decomposition through mechanisms such as direct nucleophilic substitution, Hofmann elimination, or E2 [44,45,46]. In the molecular design of AEMs, avoiding the benzyltrimethylammonium structure can significantly enhance the alkaline stability. Specifically, hexyl trimethyl ammonium has a chemical stability that is almost equivalent to that of cyclic ammonium derivatives [47].

Among the connection methods between polymer backbone and cationic group, crosslinking has been reported as a commonly used and highly effective approach to balance the ion conductivity and dimensional stability of AEMs [48,49]. Crosslinked AEMs are mainly prepared through a reaction between the backbone and the crosslinker. The reaction can be largely divided into hydrophobic crosslinking and hydrophilic crosslinking. Hydrophobic crosslinked AEMs usually possess excellent anti-swelling capabilities. However, the hydrophobic crosslinking tends to decrease the ionic conductivity, thus failing to meet the requirements of fuel cells. In contrast, hydrophilic crosslinking offers an opportunity to simultaneously limit swelling and enhance ion conductivity, and it has, therefore, received extensive research. N,N,N′,N′-tetramethyl-1,6-hexanediamine (TMHDA) is a commonly used hydrophilic crosslinker for the synthesis of AEMs through the Menshutkin reaction [50,51]. Cao et al. prepared a series of polyolefin-based AEMs through the crosslinking reaction of ethylene copolymer and propylene copolymer with TMHDA. These crosslinked AEMs exhibited high hydroxide conductivity (143 mS cm^−1^ at 80 °C) and excellent alkaline stability without visible loss in hydroxide conductivity after 1680 h [52]. Al Munsur et al. prepared a series of poly(styrene-*b*-ethylene-*co*-butylene-*b*-styrene) SEBS-based AEMs by TMHDA in which the ion conductivity increased with increasing degree of crosslinking. When chemical crosslinkers were introduced at high ratios (80%), these membranes exhibited the highest ionic conductivity (174.8 mS cm^−1^ at 80 °C) and the maximum power density of 315 mW cm^−2^ [53]. The research results by Chen et al. indicated that each polymer has an optimal degree of crosslinking to achieve the highest ionic conductivity [33]. Therefore, sufficient attention should be given to molecular design to optimize the crosslinked membrane system. In addition, the introduction of long alkoxy chains between the main chain and side chain forms a hydrogen bond network that can improve the ion conductivity and chemical stability of AEMs. Li et al. [21] designed a series of imidazolium-functionalized AEMs with varying side chain structures to investigate the effect of side chain position on AEM performance. It was found that the introduction of alkoxy chains facilitates microphase separation, thereby resulting in higher conductivity at similar IEC values and enhancing the alkaline stability. Sung et al. [54] prepared a novel poly(2,6-dimethyl-1,4-phenylene oxide) PPO-based using hydrophilic crosslinkers that contain ethylene oxide (EO), and systematically investigated the effect of crosslinker EO length. It was found that the introduction of alkoxy groups can significantly improve the ion conductivity of AEMs, and ionic conductivity initially increased and then decreased with the increase in the number of EO groups. Therefore, long alkoxy chains constructed through molecular structure design are an important approach to enhancing the ionic conductivity of AEMs.

Inspired by these prior strategies of AEMs molecular design, we designed and prepared a series of diamine crosslinked addition-type diblock poly(norbornene)s-based AEMs, which combine strategies such as an all-hydrocarbon diblock copolymer backbone, hydrophilic crosslinking, and a long alkoxy chain structure. The long alkoxy chains combined with the diblock copolymer backbone significantly boost the mobility of side chains, which is conducive to the construction of highly efficient ion transport channels. The all-hydrocarbon backbone integrated with diamine cation functional groups was used to restrain the OH^−^ attack effectively to enhance the alkaline stability; it formed crosslinked AEMs having the microphase separation structures to improve ionic conductivity, which contributed to ameliorating the “trade-off” between hydroxide conductivity and alkaline stability. Based on these structural design strategies, the regulatory mechanism of the diamine about the performances of CLx-aPNB-TMHDA-TMA AEMs (hydroxide conductivity, water uptake, swelling ratio, microphase separation morphology, chemical and thermal stabilities, and single-cell performance) was systematically investigated.

## 2. Experimental

### 2.1. Materials

5-norbornene-2-methanol (99%), sodium hydride (NaH, 60%), 1,6-dibromohexane (98%), 1-bromohexane (99%), allylpalladium chloride dimer ([(η^3^-allyl)PdCl]_2_, 98%), triphenylphosphine (PPh_3_, 99%), lithium tetrakis (pentafluorophenyl)borate-ethyl ether complex (Li[FABA], 70%), N,N,N′,N′-Tetramethyl-1,6-hexanediamine (TMHDA, 98%), trimethylamine (TMA, 35 wt% in H_2_O), trimethylamine (TMA, 2 mol/L in THF), benzotrifluoride (99%), and dichloromethane (CH_2_Cl_2_, 99.9%, Extra Dry, Water ≤ 30 ppm (by K.F.)) were purchased form Energy Chemical Co., Ltd. Tetrahydrofuran (THF, AR), ethyl acetate (AR), petroleum ether (AR), toluene (AR), acetonitrile (CH_3_CN, AR), methanol (AR), chloroform (CHCl_3_, AR), and other solvents used in this work were commercially obtained. THF and Toluene were dehydrated and deoxygenated by reflux distillation in the presence of Na/benzophenone under N_2_ atmosphere.

### 2.2. Synthesis of NB-O-Br Monomer

The synthesis of NB-O-Br was improved based on our previous work (Scheme S1a) [34]. Typically, NaH (12 g, 0.3 mol) and THF (350 mL) were added to a round-bottomed flask. Under N_2_ atmosphere, 5-norbornene-2-methanol (31.0 g, 0.25 mol) was slowly added dropwise to the flask by a constant pressure dropping funnel, and the mixture was stirred for 2 h at −78 °C under liquid nitrogen atmosphere; it then rose to 25 °C and reacted for another 24 h until no gas was generated. Further, 1,6-dibromohexane (61.0 g, 0.25 mol) was injected into the reaction flask and the reaction continued to be stirred for 24 h at 25 °C; the color of the solution changed from light gray to dark yellow. The progress was continually monitored by thin-layer chromatography (TLC) to ensure a sufficient reaction occurred. After the reaction was completed, the reaction mixture was filtered to remove sodium bromide, and the filtrate was collected. The dark yellow viscous crude product was obtained by rotary evaporation. Then, the crude product was purified by column chromatography to remove by-products and unreacted raw materials. Petroleum ether and ethyl acetate were used as eluent and developing agent, respectively, and the volume ratio (*V*_p_/*V*_e_) was 15:1. After purification, the solvent was removed via rotary evaporation under reduced pressure to obtain a colorless and transparent viscous liquid product NB-O-Br monomer.

### 2.3. Synthesis of NB-O-Hex Monomer

The synthesis of NB-O-Hex was improved based on our previous work (Scheme S1b) [14]. Typically, NaH (12 g, 0.3 mol) and THF (350 mL) were added to a round-bottomed flask. Under N_2_ atmosphere, 5-norbornene-2-methanol (31.0 g, 0.25 mol) was slowly added dropwise to the flask by a constant pressure dropping funnel; the mixture was stirred for 2 h at −78 °C, and the reaction continued at 25 °C for 24 h until no gas was produced. Further, 1-bromoheptane (41.3 g, 0.25 mol) was injected into the reaction flask and the reaction continued to be stirred for 24 h at 25 °C, until the color of the solution changed from light gray to dark yellow. Similarly, the reaction progress was tracked by TLC to ensure a complete reaction occurred. After the reaction was completed, the solution was filtered, collected, and evaporated to obtain the dark yellow viscous crude product. Then, the crude product was purified by column chromatography to remove by-products and unreacted raw materials. Petroleum ether and ethyl acetate were used as the eluent and developing agent, respectively, and the volume ratio (*V*_p_/*V*_e_) was 7:1. After purification, the solvent was removed by a rotary evaporator to obtain the colorless and transparent viscous liquid product NB-O-Hex monomer.

### 2.4. Synthesis of Allyl Palladium Chloride Complex Catalyst ((η^3^-allyl)Pd(Cl)PPh_3_)

The synthesis of (η^3^-allyl)Pd(Cl)PPh_3_ catalyst followed the approach outlined in our previous work (Scheme S1c) [14]. Allylpalladium chloride dimer [(η^3^-allyl)PdCl]_2_ (1.5 g, 4.1 mmol) and ultra-dry dichloromethane (15 mL) were added to a 100 mL round-bottomed flask that had been dewatered and deoxygenated by infrared light, and magnetically stirred for 1 h at room temperature to obtain a homogeneous solution. Triphenyl-phosphine (PPh_3_) (2.15 g, 8.2 mmol) was added to another flask and dissolved in 15 mL of ultra-dry dichloromethane. Subsequently, the [(η^3^-allyl)PdCl]_2_ solution was transferred to the PPh_3_ solution, and the reaction was stirred 24 h at room temperature under N_2_ atmosphere. The reaction mixture was concentrated to obtain a dark green powdery solid product (η^3^-allyl)Pd(Cl)PPh_3_ catalyst.

### 2.5. Synthesis of Addition-Type Norbornene-Based Diblock Copolymer (aP(NB-O-Br-b-NB-O-Hex))

**Scheme 1:**
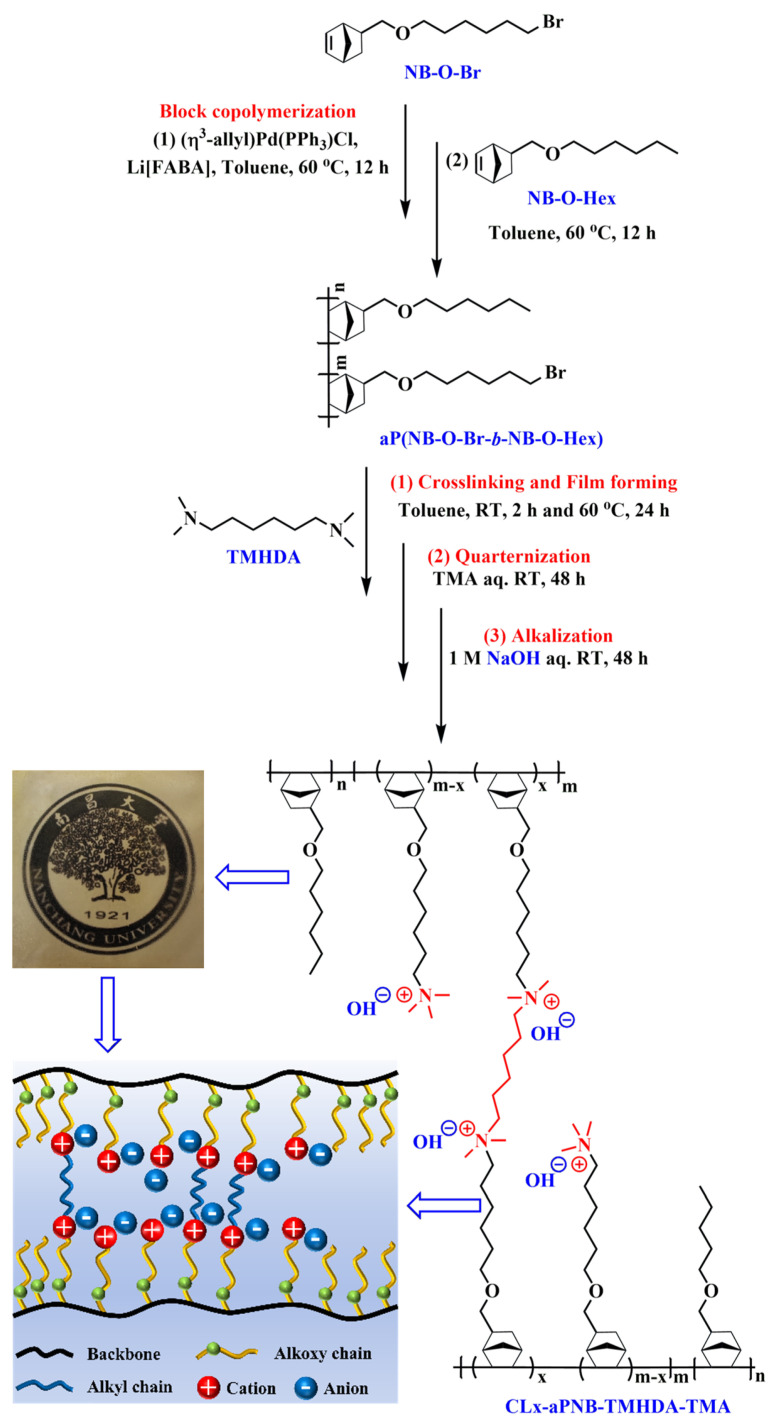
Synthetic routes of block copolymers and diamine crosslinked AEMs.

The diblock copolymer aP(NB-O-Br-*b*-NB-O-Hex) is synthesized through the step-wise vinyl addition polymerization of NB-O-Br and NB-O-Hex monomers [34]. (η^3^-allyl)Pd(Cl)PPh_3_ catalyst (32.3 mg, 0.08 mmol), Li[FABA] co-catalyst (60.8 mg, 0.08 mmol), dried toluene (5 mL), and ultra-dry trifluorotoluene (2 mL) were sequentially added to a dehydrated and deoxygenated round-bottomed flask, and then stirred for 30 min at room temperature to obtain the catalyst solution. Subsequently, NB-O-Br monomer (2.87 g, 10 mmol) was injected into the catalyst solution and stirred for 12 h at 60 °C under N_2_ protection until the first block polymerization was completed. Then, NB-O-Hex (2.08 g, 10 mmol) was injected and reacted for another 12 h at 60 °C to ensure complete second block polymerization. The reaction was then quenched with CH_3_CN. Subsequently, the reaction mixture was precipitated three times with excess methanol (200 mL). The obtained brown viscous precipitate was collected and dried under vacuum at 60 °C for 48 h to obtain the addition-type norbornene-based diblock copolymer aP(NB-O-Br-*b*-NB-O-Hex), which is termed aPNB. The synthesis route is shown in Scheme 1.

### 2.6. Preparation of Diamine Crosslinked Addition-Type Diblock Poly(Norbornene)s-Based AEMs (CLx-aPNB-TMHDA-TMA)

The equal amounts of aP(NB-O-Br-*b*-NB-O-Hex) were separately added into four 10 mL sample bottles, and toluene was used as the solvent to prepare 5 wt% copolymer solutions. Subsequently, TMHDA crosslinking agent was added to each copolymer solution at concentrations of 10%, 20%, 30%, and 40% (relative to the molar content of halogen groups in the copolymer), and the mixtures were stirred at room temperature for 2 h. The homogeneous film-forming solution was obtained by filtration through a 0.45 μm PTFE filter membrane. The solution was then cast into a PTFE (polytetrafluoroethylene) mold and vacuum-dried at 60 °C for 24 h to ensure completion of the crosslinking reaction. The membranes were peeled off the PTFE mold and immersed in TMA at room temperature for 48 h to quaternize the bromohexyl head groups. The quaternized membrane in Br^−^ form was washed thoroughly with DI water and soaked in 1 M NaOH for 48 h under N_2_ to exchange the Br^−^ for OH^−^. The crosslinked AEMs were denoted as CLx-aPNB-TMHDA-TMA, where x means the degree of crosslinking of the AEMs. The synthetic route is shown in Scheme 1.

### 2.7. Preparation of Non-Crosslinked Addition-Type Diblock Poly(Norbornene)s-Based AEMs (aPNB-TMA)

The non-crosslinked addition-type diblock poly(norbornene)-based AEMs were prepared for comparison. Referring to the preparation method of CLx-aPNB-TMHDA-TMA, a 5 wt% copolymer solution was prepared and then filtered through a 0.45 μm PTFE membrane to obtain the homogeneous film-forming solution. The membranes were peeled off the PTFE mold and immersed in TMA at room temperature for 72 h to quaternize the bromohexyl head groups. The quaternized membrane in Br^−^ form was washed thoroughly with DI water and soaked in 1 M NaOH for 48 h under N_2_ to exchange the Br^−^ for OH^−^. The non-crosslinked AEMs were denoted as aPNB-TMA.

### 2.8. Characterization and Measurements

All the detailed characterization and measurement methods are described in the Supporting Information.

## 3. Results and Discussion

### 3.1. Structure Analysis

The NB-O-Br and NB-O-Hex monomers were successfully synthesized via Williamson etherification reaction, as shown in Scheme S1 [14,34]. Their chemical structures were confirmed by ^1^H NMR (Figure S1) and FTIR (Figure S2) spectrums, respectively. It was evident that all signals in the ^1^H NMR spectrum were consistent with the corresponding molecular structure and indicated that the NB-O-Br and NB-O-Hex monomers had been successfully synthesized. Additionally, their chemical structures were further confirmed by FTIR spectroscopy, where the characteristic peak at 3055 cm^−1^ was attributed to the = C-H bond on the norbornene ring. The characteristic absorption peak at 1105 cm^−1^ was related to the ether bond on the side chain. The combined analysis of ^1^H NMR and FTIR spectra collectively confirmed the successful synthesis of these two monomers.

**Figure 1:**
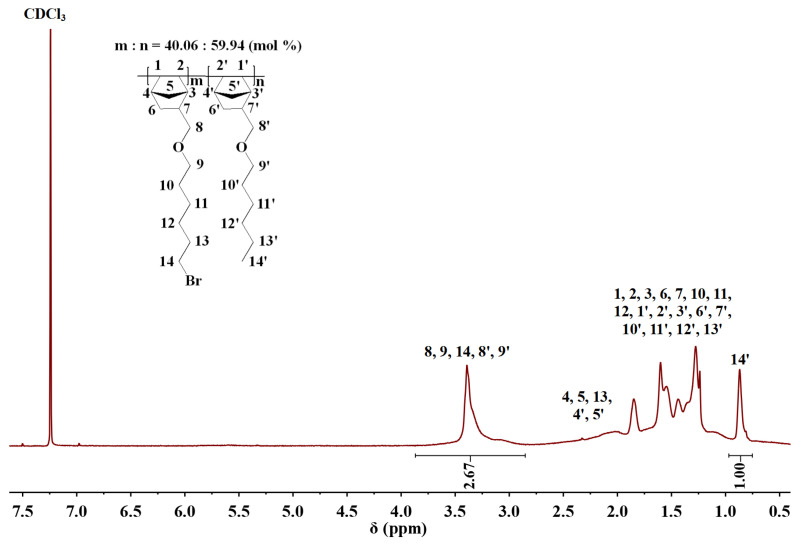
^1^H NMR spectra of aP(NB-O-Br-*b*-NB-O-Hex).

**Figure 2:**
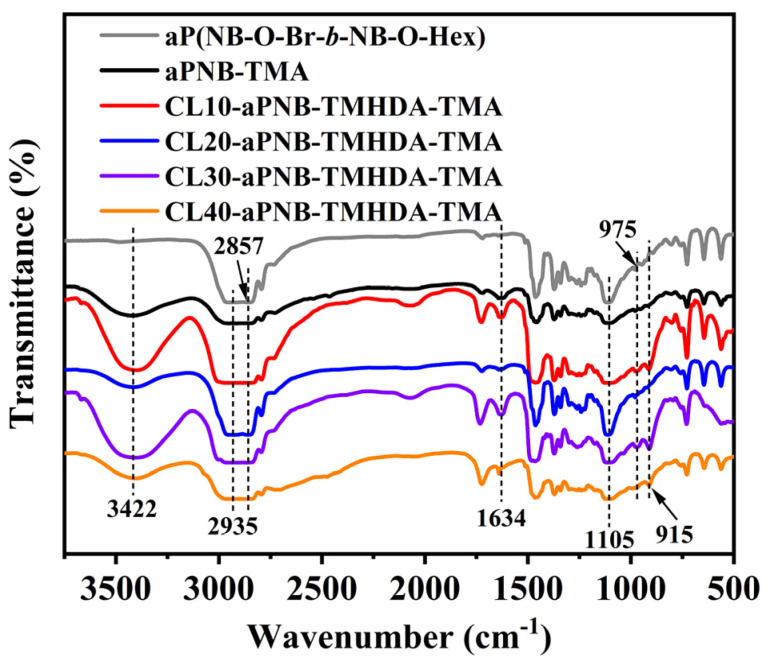
FTIR spectra of aP(NB-O-Br-*b*-NB-O-Hex), aPNB-TMA and CLx-aPNB-TMHDA-TMA.

The diblock copolymer aP(NB-O-Br-*b*-NB-O-Hex) was synthesized by polymerization of NB-O-Br and NB-O-Hex catalyzed by the late transition metal catalyst system (η^3^-allyl)Pd(Cl)PPh_3_/Li[FABA] [34], and its chemical structure was verified by ^1^H NMR (Figure 1) and FTIR spectra (Figure 2). In the ^1^H NMR spectra of aP(NB-O-Br-*b*-NB-O-Hex), the signals at 2.94–3.89 ppm were attributed to the methylene groups connected to the ether bond and bromine functional group (H^8^,H^8′^,H^9^,H^9′^,H^14^), and the peak at 0.88 ppm was associated with the methyl group at the end of the ether chain (H^14′^) [34]. The ratio of proton peak areas A_(H8,H8′,H9,H9′,H14)_/A_(H14′)_ was calculated to be 2.67, from which the ratio of the amount of substance of NB-O-Br to NB-O-Hex in the diblock copolymer was determined to be 40.06:59.94. Meanwhile, there was no characteristic signal near 5.25 ppm, which was attributed to the C = C bonds on the main chain of ROMP-type norbornene polymers [28]. The positions and shapes of these characteristic peaks both indicate the successful preparation of the diblock copolymer aP(NB-O-Br-*b*-NB-O-Hex). Furthermore, the absorption peaks observed at approximately 3055 cm^−1^ and in the 1571–1622 cm^−1^ region were ascribed to the = C-H and C = C bonds in the norbornene rings of the NB-O-Br and NB-O-Hex monomers, respectively. The complete disappearance of both peaks following copolymerization indicates the sufficient progress of the vinyl addition polymerization reaction.

The crosslinked AEMs CLx-aPNB-TMHDA-TMA were characterized by FTIR spectroscopy (Figure 2), as they were insoluble in deuterated reagents and, therefore, could not be characterized by NMR. The chemical structure of the non-crosslinked AEM aPNB-TMA was also confirmed by FTIR spectroscopy for comparison. The absorption peaks at 2935 cm^−1^ and 2857 cm^−1^ were ascribed to the methylene groups [28]. All AEMs exhibited new characteristic peaks at 915 cm^−1^, 975 cm^−1^, and 1634 cm^−1^, which were related to the quaternary ammonium cation groups [31,52]. The broad band at ~3422 cm^−1^ was attributed to the O-H stretching vibration of the absorbed moisture due to hydrophilicity of the AEMs [34,51]. Thus, the FTIR results confirmed the successful preparations of diamine crosslinked AEMs CLx-aPNB-TMHDA-TMA.

### 3.2. Solubility of Copolymers and Diamine Crosslinked AEMs

The solubility of diblock copolymers and crosslinked AEMs was tested, and the crosslinking degree of CLx-aPNB-TMHDA-TMA membranes was characterized through calculating the gel fraction (GF) of AEMs. As shown in Table 1, the aP(NB-O-Br-*b*-NB-O-Hex) copolymer exhibited good solubility in THF, CHCl_3_, toluene, and chlorobenzene, while the CLx-aPNB-TMHDA-TMA membrane was insoluble in these solvents. This can be attributed to the crosslinking reaction that occurred between the copolymer and TMHDA, resulting in a covalent crosslinked network structure that enhances the solubility resistance of the AEMs. This was confirmed by the ^1^H NMR spectrum of the solution obtained after the GF test (Figure S3). Furthermore, the GF values of CLx-aPNB-TMHDA-TMA were all above 75%, and they increased with the increasing amount of hydrophilic crosslinker TMHDA used. Among them, CL40-aPNB-TMHDA-TMA exhibited the highest GF of 90.12%, indicating the highest degree of crosslinking.

### 3.3. Morphology Characterization

**Figure 3:**
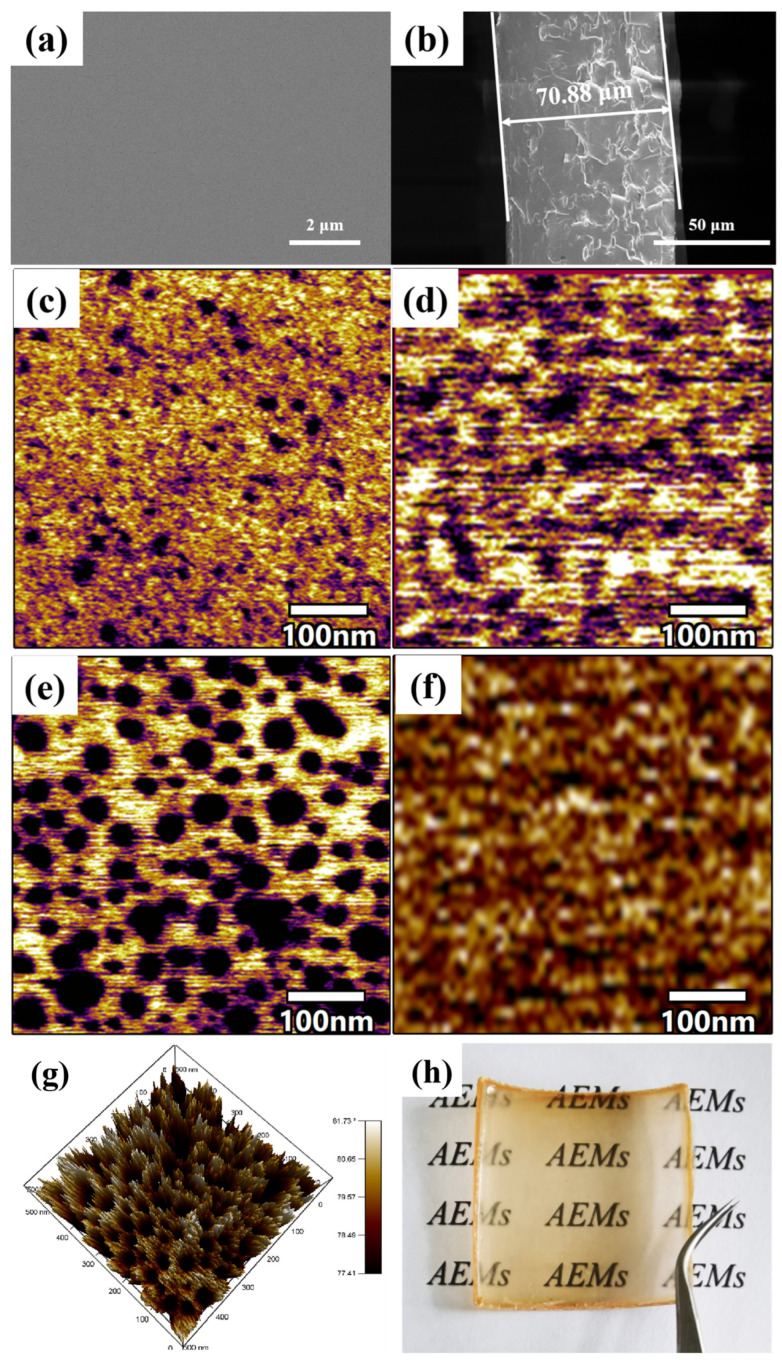

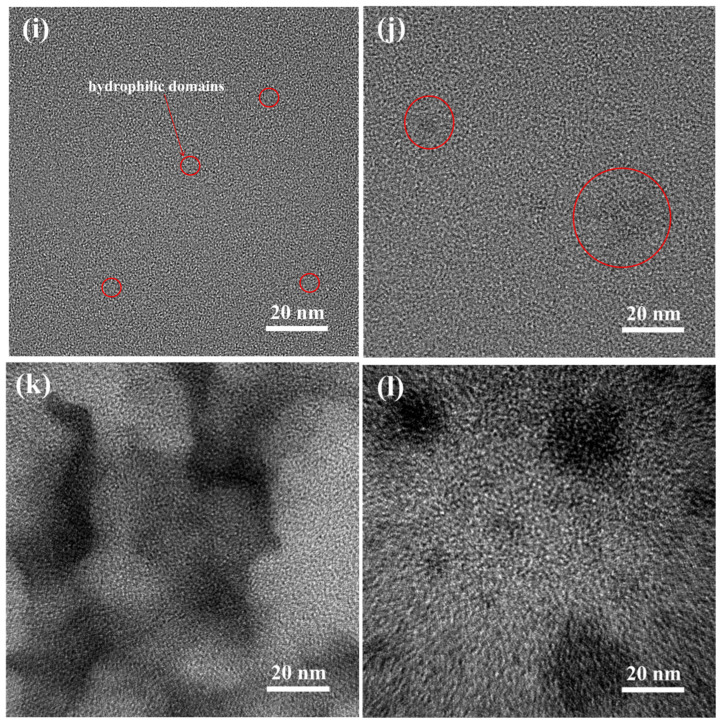
SEM images of CL30-aPNB-TMHDA-TMA: (**a**) surface and (**b**) cross-section; AFM images of (**c**) CL10-aPNB-TMHDA-TMA, (**d**) CL20-aPNB-TMHDA-TMA, (**e**) CL30-aPNB-TMHDA-TMA, (**f**) CL40-aPNB-TMHDA-TMA, (**g**) AFM 3D image of CL30-aPNB-TMHDA-TMA; and (**h**) digital photograph of CLx-aPNB-TMHDA-TMA membrane; and TEM of (**i**) CL10-aPNB-TMHDA-TMA, (**j**) CL20-aPNB-TMHDA-TMA, (**k**) CL30-aPNB-TMHDA-TMA, and (**l**) CL40-aPNB-TMHDA-TMA.

The morphology of the crosslinked CL30-aPNB-TMHDA-TMA membrane was observed by SEM. The surface and cross-section SEM images of the crosslinked CL30-aPNB-TMHDA-TMA membrane are shown in Figure 3a,b. A smooth and uniform structure with approximately 70.88 μm in thickness was clearly observed. The SEM cross-sectional image of CL30-aPNB-TMHDA-TMA showed no defects such as penetrating holes or cracks, effectively preventing fuel penetration. Furthermore, the CLx-aPNB-TMHDA-TMA membranes were light yellow, smooth, and dense at the macroscopic level (Figure 3h). The smooth surface of the AEMs will enhance an effective contact leading to a high catalytic efficiency. Therefore, the aPNB-based crosslinked membranes can exhibit good performances in AEMFCs due to their high catalytic efficiency and their good fuel resistance.

The construction of ion-conducting channels in AEMs via hydrophilic/hydrophobic microphase separation is believed to be one of the most effective strategies to improve the ion conductivity [47]. The microphase separation morphology of membranes was measured by AFM to intuitively display the microstructures in CLx-aPNB-TMHDA-TMA and the results are shown in Figure 3c–g. In the AFM images, dark regions refer to hydrophilic domains composed of cationic clusters, while light regions correspond to hydrophobic domains formed from polymer backbones. It can be easily observed that as-prepared AEMs all reveal distinct microphase separation. Meanwhile, as the amount of TMHDA increased, the size of hydrophilic clusters (dark regions) exhibited a trend of initially increasing and then decreasing, with the CL30-aPNB-TMHDA-TMA membrane possessing the largest hydrophilic areas and continuous ion transport pathways (Figure 3g). Moreover, the TEM was used to further characterize the microphase separation structure of AEMs, as illustrated in Figure 3i–l. Similarly, the TEM images of CLx-aPNB-TMHDA-TMA exhibited consistent results with those observed in the AFM phase images. The darker areas represent the aggregation domains of the hydrophilic cations, whereas the brighter areas correspond to the aggregation domains of the hydrophobic copolymer backbone portion. This is potentially attributed to the balance between the promoting effect of the flexible alkoxy structure on the movement of long side chains and the crosslinking effect of hydrophilic crosslinkers. At low crosslinking degrees, the flexible alkoxy groups exhibited a strong promoting effect on the movement of long side chains, whereas the crosslinking action of hydrophilic crosslinkers was relatively weak, leading to increased interchain entanglement among polymer chains, which hindered the formation of microphase separation structures within the membrane. Conversely, at excessively high crosslinking degrees, the mobility of cationic groups decreased while the crosslinking effect became dominant, impeding the self-aggregation of ionic clusters and consequently resulting in a reduction in the size of hydrophilic clusters. Therefore, it is considered that there is an optimal degree of crosslinking for each polymer backbone, which enables the prepared membrane to exhibit the best microphase separation morphology [33].

**Figure 4:**
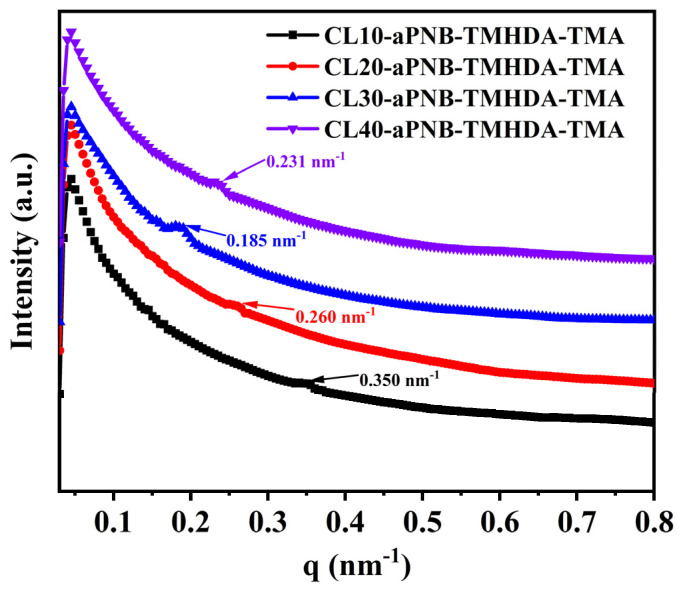
SAXS spectra of CLx-aPNB-TMHDA-TMA.

The CLx-aPNB-TMHDA-TMA membranes were further analyzed by SAXS for morphologies and the results are shown in Figure 4. In the figure, there were slight peaks at 0.350, 0.260, 0.185, and 0.231 nm^−1^ for CL10-aPNB-TMHDA-TMA, CL20-aPNB-TMHDA-TMA, CL30-aPNB-TMHDA-TMA, and CL40-aPNB-TMHDA-TMA, respectively. Correspondingly, the inter-domain spacing (*d*) was calculated to be 17.95, 24.17, 33.96, and 27.20 nm according to the Bragg equation (*d* = 2*π q*^−1^) [10]. This indicates that distinct microphase separation was formed in the CLx-aPNB-TMHDA-TMA AEMs, with the CL30-aPNB-TMHDA-TMA AEM exhibiting the largest ion domain. The results were in agreement with the AFM characterization, and collectively confirmed that the prepared AEMs possess well-connected ion transmission pathways.

### 3.4. Ion Exchange Capacity, Water Uptake, Swelling Ratio, and Hydration Number

As a key parameter, ion exchange capacity (IEC) plays a critical role in water uptake (WU), swelling ratio (SR), and conductivity of AEMs to some extent. A reasonable WU is crucial for conductivity during the process of ion transport. A low WU limits ion migration, but excess water dilutes OH^−^ when WU is sufficiently high. Further, excess water leads to severe swelling, which affects the stability of membranes [55,56]. As listed in Table 2, the prepared membranes exhibited similar IECs determined by titration, and these titrated IECs were consistent with the theoretical IECs, revealing that the grafting ratio of the quaternary ammonium cation groups in these membranes approximates the monomer ratio. Moreover, it was evident that the hydration number (λ) of the resulting AEMs decreased with increasing crosslinking degree. It can be reasonably assumed that the crosslinked network structure increases the interactions among copolymer chains and, therefore, restricts the free volume, which inhibits excessive water uptake of the membranes.

**Figure 5:**
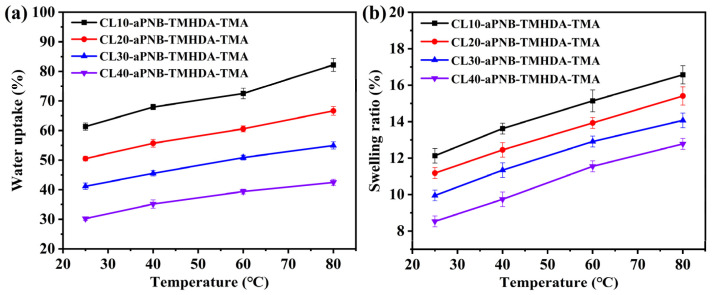

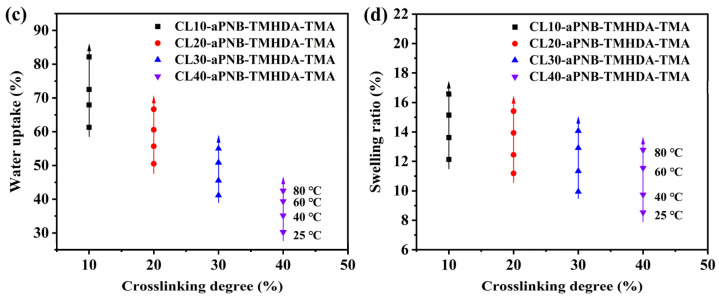
(**a**) WU and (**b**) SR as a function of temperatures; (**c**) WU and (**d**) SR as a function of crosslinking degrees for CLx-aPNB-TMHDA-TMA.

Further, the water uptake (WU) and swelling ratio (SR) of the four crosslinked aPNB-based membranes were measured to investigate the influence of hydrophilic crosslinker content on the water absorption behavior of CLx-aPNB-TMHDA-TMA membranes. Notably, the non-crosslinked aPNB-TMA membrane was highly prone to swelling and rupturing at 60 °C and 80 °C, which subsequently prevented the completion of further testing for WU, SR, and ion conductivity (Figure S4). As plotted in Figure 5a,b, the WU was in the range of 30.21% to 61.33%, and the SR was in the range of 8.53% to 12.13% at 25 °C. As expected, both the WU and SR increased with the rise in temperature, reaching 42.49–82.18% and 12.78–16.57%, respectively, at 80 °C, which was primarily attributed to the strengthening of mobility of water molecules and polymer segments as temperature increased. In addition, the WU and SR of CLx-aPNB-TMHDA-TMA membranes decreased with the increase in crosslinking degree (Figure 5c,d), indicating that the crosslinking strategy can enhance the interaction between polymer chains and improve the water retention ability of the membranes, allowing them to maintain a high water uptake with low swelling [31,49]. Therefore, it is concluded that increasing the crosslinking degree by adding hydrophilic crosslinkers to limit excessive swelling of AEMs is an effective strategy to enhance dimensional stability and maintain ion exchange capacity.

### 3.5. Ionic Conductivity

**Figure 6:**
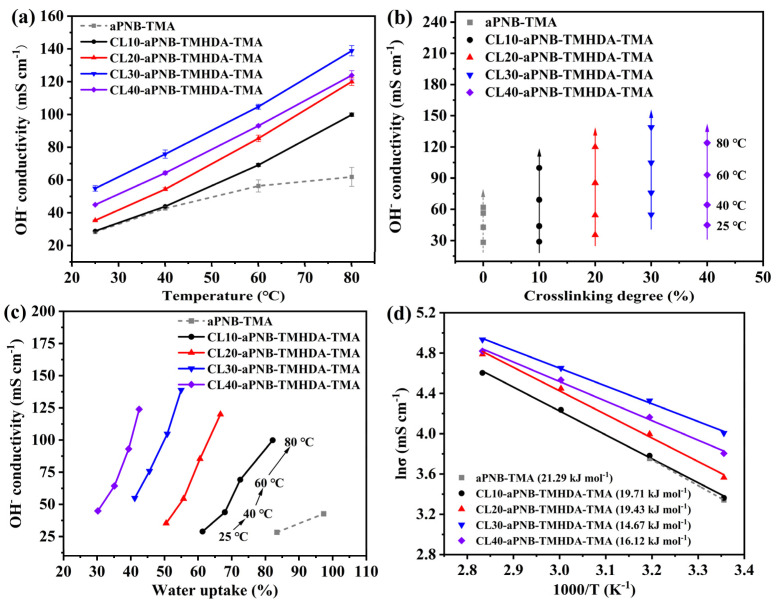
OH^−^ conductivity as a function of (**a**) temperatures and (**b**) crosslinking degrees for CLx-aPNB-TMHDA-TMA and aPNB-TMA membranes, (**c**) OH^−^ conductivity as a function of WU for CLx-aPNB-TMHDA-TMA membranes and (**d**) the Arrhenius plots of CLx-aPNB-TMHDA-TMA membranes.

The hydroxide ion conductivity (σ) is one of the key properties for evaluating the application performance of AEMs, and it directly affects the application of the membrane. Hydroxide conductivities of all AEMs are compared in Figure 6a. In the case of crosslinked aPNB-based membranes with the TMHDA crosslinker, the hydroxide ion conductivity was measured to be 28.86 to 54.86 mS cm^−1^ at 25 °C, and 99.82 mS cm^−1^ to 138.84 mS cm^−1^ at 80 °C. In the case of non-crosslinked aPNB-based membranes, the hydroxide ion conductivity was measured to be 28.28 mS cm^−1^ at 25 °C and 61.88 mS cm^−1^ at 80 °C (swelling and rupturing occurred at 60 °C and 80 °C). It was obvious that the OH^−^ conductivity of AEMs increased with temperature. This is because as the temperature rose, the migration rate of OH^−^ increased, and the movement of polymer segments within the membrane accelerated, which together promoted the conduction of OH^−^. Moreover, the OH^−^ conductivity of all AEMs exhibited a trend of an initial increase followed by a decrease as the crosslinking degree increased at the same temperature (Figure 6b). Notably, the CL30-aPNB-TMHDA-TMA membrane exhibited the highest OH^−^ conductivity, reaching 138.84 mS cm^−1^ at 80 °C, which can be attributed to the balance between the promotion of cationic group mobility by the alkoxy flexible long side chain structure and the inhibition of OH^−^ conductivity by the crosslinked structure. At the low crosslinking degrees, the membranes exhibited a relatively high WU, leading to “dilution” of OH^−^ ions and narrower ion transport channels within the membranes [57]. Conversely, at the high crosslinking degrees, the membrane’s WU decreased, resulting in a “concentration” of OH^−^ ions and relatively wider ion transport channels within the membranes. This was also confirmed by the aforementioned AFM images and WU measurements. Further, the functional relationship between the OH^−^ conductivity of AEMs and WU is illustrated in Figure 6c. As expected, the OH^−^ conductivity of AEMs was proportional to WU, indicating that increasing WU can enhance OH^−^ conductivity to some extent. However, excessively high WU can lead to excessive swelling of the membranes. Therefore, a reasonable WU plays a crucial role in the preparation of AEMs that balance high conductivity and good dimensional stability.

The Arrhenius equation can explain the correlation of OH^−^ conductivity against temperature, and the activation energy (*E*_a_) about OH^−^ conductivity in membranes can be resultant from the slopes of the ln(σ)-1000/T graph for CLx-aPNB-TMHDA-TMA membranes, as shown in Figure 6d. It shows the activation energy for OH^−^ transport calculated by the Arrhenius equation. The activation energy of the CL30-aPNB-TMHDA-TMA membrane was lower than that of the others. The lower activation energy reduced the ion transport energy barrier, and it contributed to the directional transfer of OH^−^, further demonstrating that the channels constructed with the appropriate crosslinking density can enhance OH^−^ transport.

### 3.6. Mechanical Properties and Thermal Stability

**Figure 7:**
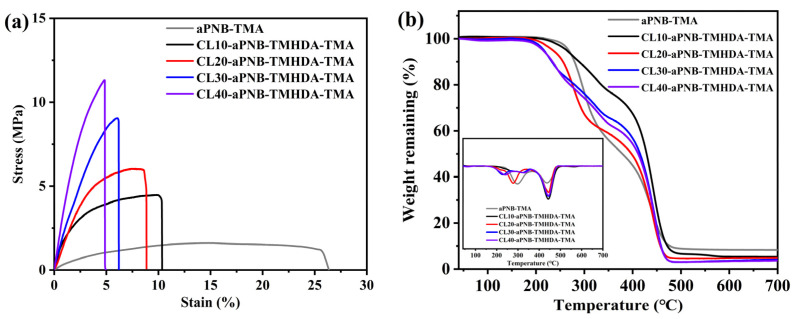
(**a**) The mechanical properties of CLx-aPNB-TMHDA-TMA and aPNB-TMA membranes, (**b**) the TGA curves of CLx-aPNB-TMHDA-TMA and aPNB-TMA membranes with the inset showing DTG curves.

The mechanical properties of AEMs are critical for assembling robust MEAs and long-term stability of membranes for fuel cells. As shown in Figure 7a, the tensile strength and elongation at break of the crosslinked AEMs were in the range of 4.48–11.31 MPa and 4.92–10.35%, respectively. In contrast, the tensile strength of the non-crosslinked membrane was 1.61 MPa, and the elongation at break was 26.35%. Compared to non-crosslinked aPNB-TMA membranes, increased tensile strength and decreased elongation at break of the fabricated membrane is attributed to the crosslinked network, improving the tensile strength of the membrane and limiting the movement of the polymer chains [34,48]. Furthermore, the tensile strength of the membranes increased with an increase in the crosslinking degree for the crosslinked membranes. Therefore, hydrophilic crosslinking is an effective strategy to enhance the mechanical properties of AEMs. The prepared AEMs can provide sufficient mechanical strength for fuel cell applications.

Thermal stability is one of the crucial factors that determine the service life of fuel cells. The thermal stability of as-prepared crosslinked membranes (CLx-aPNB-TMHDA-TMA) and non-crosslinked membranes (aPNB-TMA) were measured via thermogravimetric analysis (TGA). As shown in Figure 7b, the TGA and DTG curves of the CLx-aPNB-TMHDA-TMA membranes displayed four degradation stages. The first weight loss occurred at approximately 150 °C and was caused by the evaporation of the water or solvents remaining on the polymer membranes. The second degradation stage, at around 200–285 °C, was related to the thermal degradation of quaternary ammonium groups and crosslinked structures. Further, the third weight loss, occurring at 285–375 °C, was due to the degradation of the long side chains. Lastly, the weight loss that occurred after 375 °C corresponded to degradation of the remainder of the polymer backbone. However, the non-crosslinked aPNB-TMA membrane exhibited only three degradation stages in its TGA and DTG curves. The first and third weight loss stages were analogous to those observed in the crosslinked membranes, whereas the second degradation stage, occurring at approximately 200–375 °C, was attributed to the degradation of the quaternary ammonium groups and long side chains. Given that the typical operating temperature of fuel cells falls within the range of 60–80 °C, the prepared AEMs exhibited good thermal stability at temperatures below 200 °C. Therefore, the CLx-aPNB-TMHDA-TMA membranes have excellent thermal stability that can meet the application of fuel cells completely.

### 3.7. Alkaline and Oxidative Stability

**Figure 8:**
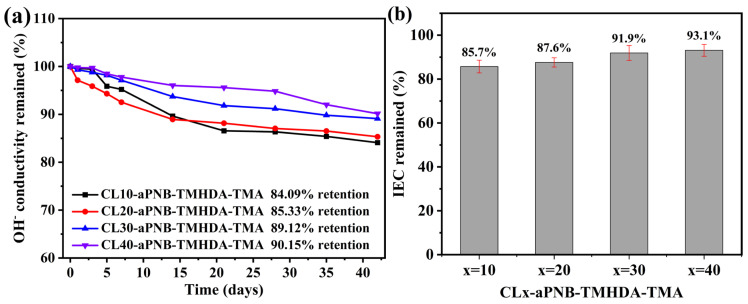
(**a**) The remaining hydroxide conductivity and (**b**) the remaining IEC at 80 °C after alkaline stability test.

Long-term stability of the AEMs under highly alkaline conditions is critical. It is known that degradation mechanisms of quaternary ammonium are Hofmann elimination and nucleophilic substitution [46]. In this work, in an attempt to evaluate the alkaline stability of the membranes, their OH^−^ conductivity was examined in a 1 M aqueous NaOH solution at 80 °C for about 1008 h at a certain time interval, in terms of the magnitude of its decrease over time. After a certain period of time, these membranes were taken out of the NaOH solution and carefully washed with DI water. The OH^−^ conductivity of each membrane was measured at 80 °C. As shown in Figure 8a, the remaining OH^−^ conductivity of the AEMs after 1008 h was in the range of 84.09–90.15%. All AEMs exhibited good alkaline stability, which improves with increasing crosslinking degree. The reason is that the crosslinked structures and the steric hindrance of the long alkoxy side chains protect the functional groups away from OH^−^ attacking [54]. In addition, benefiting from the synergistic effects of the all-carbon hydrogen block copolymer backbone, long flexible alkoxy groups, and hydrophilic crosslinked structure, a well-defined microphase separation structure is formed within the membrane. This facilitates faster conduction of OH^−^, prevents local “aggregation” of OH^−^ within the membrane, and ultimately enhances the long-term alkaline stability of the AEMs. As shown in Figure S5, compared to the previous literature, the CLx-aPNB-TMHDA-TMA membranes keep a good balance between ionic conductivity and alkaline stability than other AEMs.

**Figure 9:**
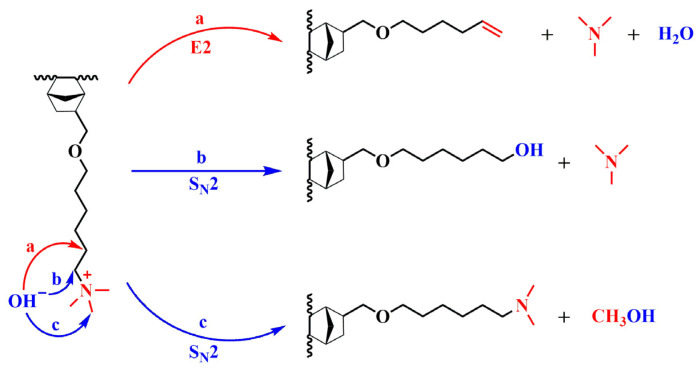
The possible degradation mechanism of quaternary ammonium cations.

Further, the IEC values of crosslinked membranes were measured after the alkaline test and were compared with those before the test. As shown in Figure 8b, the trends in IEC values were similar to those of the conductivities measured before and after the alkaline test. Both conductivity and IEC analysis results suggest that the degradation of the crosslinked membranes can mainly be ascribed to the degradation of the conducting head groups. The possible degradation mechanisms of the quaternary ammonium cationic groups are shown in Figure 9. The quaternary ammonium cationic groups undergo E2 elimination and S_N_2 substitution, ultimately resulting in the generation of tertiary amine groups that lack the ability to conduct, thus leading to a decrease in OH^−^ conductivity.

**Figure 10:**
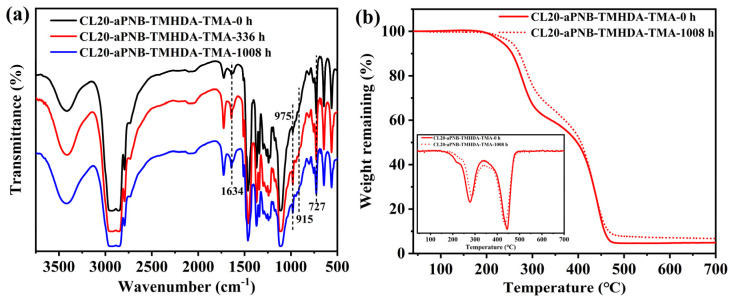
(**a**) FTIR spectra and (**b**) TGA curves of CL20-aPNB-TMHDA-TMA before and after alkaline treatment.

FTIR spectra were further used to examine the changes in the chemical structure of the membranes after the alkaline resistance test. As analyzed from Figure 10a, the stretching peaks of the quaternary ammonium groups at 915 cm^−1^, 975 cm^−1^, and 1634 cm^−1^ remained nearly unchanged, indicating that the quaternary ammonium groups have no significant degradation. Figure 10b displays the TGA and DTG curves of the crosslinked membranes before and after alkaline resistance test, and the membranes can still keep outstanding thermal stability. Furthermore, the oxidative stability of AEMs was further validated through immersion in Fenton’s reagent at 80 °C for 8 h (Figure S6), and the residual weight and conductivity after the treatment were assessed to evaluate the oxidative stability of AEMs. It is evident that CL40-aPNB-TMHDA-TMA demonstrated the highest oxidative stability, with a weight retention of 97.9% and a conductivity retention of 97.8%. In summary, the CLx-aPNB-TMHDA-TMA membranes exhibited excellent stability and thus have the potential for application in fuel cells.

### 3.8. Single Cell Performance

**Figure 11:**
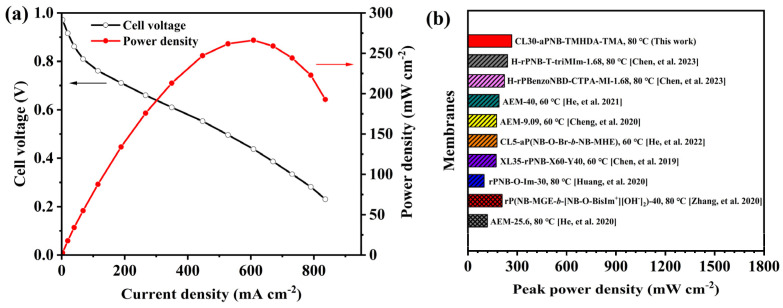
(**a**) Cell voltage and power density curves of H_2_/O_2_ fuel cell equipped with the CL30-aPNB-TMHDA-TMA AEM at 80 °C, and (**b**) comparison of the single cell performance between this work and similarly structured AEMs reported in the literature [28,31,33,34,58,59,60,61,62].

AEM plays a crucial role in ion conduction during the operation of AEMFCs. The CL30-aPNB-TMHDA-TMA AEM, with high hydroxide conductivity and excellent alkaline stability, was evaluated in a single fuel cell to examine its applicability in fuel cells. The single cell performance was determined under operation conditions of an H_2_/O_2_ feed flow rate of 400/400 mL min^−1^ at 80 °C and 100% relative humidity. The polarization curve and power density were plotted in Figure 11a. The open circuit voltage of the cell was about 0.97 V, indicating that the CL30-aPNB-TMHDA-TMA membrane has excellent gas barrier properties to separate the H_2_ and O_2_. In addition, the peak power density was 266.2 mW cm^−2^ at a current density of 608 mA cm^−2^ at 80 °C, which outperforms the reported AEMs with similar structures (Figure 11b) [28,31,33,34,58,59,60,61,62] and thus demonstrates the good fuel cell performance. However this value is far lower than the best record reported in the literature (beyond 3 W cm^−2^) [32,63,64]; it should be noted that the fuel cell performance is not only determined by AEMs, but also affected by multiple complex aspects, such as MEA preparation technology, single cell assembly methods, and testing environment [11,12,65,66]. Therefore, it is believed that the performance can be improved by optimizing MEA fabrication methods, such as catalyst-coated membranes (CCMs), as well as the operating conditions.

## 4. Conclusions

In summary, a series of tunable diamine crosslinked CLx-aPNB-TMHDA-TMA membranes were successfully prepared via the Menshutkin reaction. The resulting addition-type diblock poly(norbornene)s-based AEMs overcame the “trade-off” relationship between ion conduction and the dimensional stability of crosslinked AEMs, and thus exhibited a high hydroxide conductivity (>99.82 mS cm^−1^) and a low swelling ratio (SR < 17%). The well-developed microphase separation morphology was observed for all the crosslinked AEMs using SAXS, AFM, and TEM. In particular, the CL30-aPNB-TMHDA-TMA exhibited a high hydroxide conductivity with 54.86 mS cm^−1^ at 25 °C and 138.84 mS cm^−1^ at 80 °C, respectively. Moreover, it demonstrated excellent alkaline stability after immersion in 1 M NaOH aqueous solution at 80 °C for 1008 h, with the IEC and hydroxide conductivity remaining at 91.9% and 89.12% of their initial values, respectively. These excellent performances are attributed to the synergistic strategy that combines all-carbon hydrogen block copolymer backbones, long flexible alkoxy spacer chains, and a cationic hydrophilic crosslinking structure. Further, the H_2_/O_2_ single cell assembled with CL30-aPNB-TMHDA-TMA MEA exhibited a peak power density of 266.2 mW cm^−2^ at a current density of 608 mA cm^−2^ at 80 °C. Therefore, it can be concluded that the prepared addition-type diblock poly(norbornene)-based AEMs exhibit excellent research and development potential, making them potential candidates for AEMFCs.

## Figures and Tables

**Table 1:** The solubility and GF of (a) aP(NB-O-Br-*b*-NB-O-Hex), (b) CL10-aPNB-TMHDA-TMA, (c) CL20-aPNB-TMHDA-TMA, (d) CL30-aPNB-TMHDA-TMA, and (e) CL40-aPNB-TMHDA-TMA.

AEMs	THF	CHCl_3_	Toluene	Chlorobenzene	CH_2_Cl_2_	DMSO	GF (wt%)
a	+	+	+	+	±	−	0
b	−	−	−	−	−	−	75.37 ± 1.1
c	−	−	−	−	−	−	81.71 ± 0.8
d	−	−	−	−	−	−	86.25 ± 0.5
e	−	−	−	−	−	−	90.12 ± 0.9

+: completely soluble at room temperature; −: completely insoluble at room temperature; ±: partially soluble at room temperature.

**Table 2:** IEC, WU, SR, and λ of aPNB-TMA and CLx-aPNB-TMHDA-TMA.

AEMs	IEC (mmol g^−1^)		WU ^c^ (%)	SR ^c^ (%)	λ
	Cal. ^a^	Exp. ^b^			
aPNB-TMA	1.68	1.66 ± 0.03	83.43 ± 2.8	17.17 ± 0.8	27.92
CL10-aPNB-TMHDA-TMA	1.66	1.61 ± 0.05	61.33 ± 1.2	12.13 ± 0.4	21.16
CL20-aPNB-TMHDA-TMA	1.65	1.61 ± 0.04	50.52 ± 0.8	11.18 ± 0.3	17.43
CL30-aPNB-TMHDA-TMA	1.63	1.60 ± 0.03	41.17 ± 1.1	9.95 ± 0.3	14.30
CL40-aPNB-TMHDA-TMA	1.62	1.59 ± 0.04	30.21 ± 0.6	8.53 ± 0.3	10.56

^a^ Theoretical IEC calculated from ^1^H NMR; ^b^ Experimental IEC determined by titration; ^c^ Measured at 25 °C.

## Data Availability

The data are available on reasonable request from the corresponding author.

## Supplementary Materials

The following supporting information can be downloaded at: https://www.mdpi.com/article/10.3390/polym16243534/s1, Scheme S1: Synthesis of (a) NB-O-Br monomer, (b) NB-O-Hex monomer, and (c) (η^3^-allyl)Pd(Cl)PPh_3_ catalyst; Figure S1: ^1^H NMR spectra of (a) NB-O-Br and (b) NB-O-Hex monomer; Figure S2: FTIR spectra of 1,6-Dibromohexane, TMHDA, NB-O-Br and NB-O-Hex; Figure S3: ^1^H NMR spectra of the residues in the solution after the GF test of AEM; Figure S4: The photograph of the swelling and rupture of the non-crosslinked membrane observed during the swelling ratio test at 40 °C; Figure S5: Alkaline stability and conductivity of the membranes in this work and the literature; Figure S6: Oxidative stability of the prepared AEMs immersed in Fenton’s solution (4 ppm Fe^2+^ in 3 wt% H_2_O_2_) at 80 °C (remaining weight and conductivity); Figure S7: ^1^H NMR spectra of aPNB-TMA-1.68 ionomer. Refs. [13,14,31,33,34,54,58,59,60,66] are cited in the Supplementary Materials.
