# Supplementary material for: Diamine Crosslinked Addition-Type Diblock Poly(Norbornene)s-Based Anion Exchange Membranes with High Conductivity and Stability for Fuel Cell Applications
Source: Polymers (Basel). 2024 Dec 18;16(24):3534. doi: 10.3390/polym16243534 (PMC11677948; doi:10.3390/polym16243534)
Supplement: Supplementary file 1 [file polymers-16-03534-s001.zip › polymers- 3342402 -Supporting Information.pdf]

Supporting Information

Diamine crosslinked addition-type diblock poly(norbornene)s-based anion exchange membranes with high conductivity and stability for fuel cell applications

Quan Li[a], Xiaohui He[a]*, Ling Feng[a], Jia Ye[a], Wenjun Zhang[a], Longming Huang[a], Defu Chen[b]

[a]School of Physics and Materials Science, Nanchang University, 999 Xuefu Avenue, Nanchang 330031, China

[b]School of Civil Engineering and Architecture, Nanchang University, 999 Xuefu Avenue, Nanchang 330031, China

*Correspondence to: X. He (E-mail: hexiaohui@ncu.edu.cn)

1. Characterization and measurements

1.1 Chemical structure

The ^1H NMR spectra were characterized by the Bruker ARX600 Nuclear Resonance Spectrometer (400 MHz, Switzerland). The internal standard was tetramethylsilane (TMS), and $CDCl_3$ or DMSO-d_6 were used as the solvent. Fourier Transform Infrared (FIIR) measurements were collected on an IR Prestige-21 FT-IR spectrometer (Shimadzu, Japan) in the range of 4000-500 cm^{-1}.

1.2 Gel fraction (GF)

Gel fraction was measured by soaking the membrane in chloroform for 72 h at 60 °C, then treated by vacuum drying at 60°C for 48 h. The gel fraction (GF) was calculated by Eq. (1):

$$Gel\,fraction = \frac{W_s}{W_a} \times 100\% \qquad (1)$$

where W_a and W_s are the weight of dry membrane specimen before and after immersion in chloroform, respectively.

1.3 Morphology

The morphology of AEMs was conducted by Scanning electron microscopy (SEM, ZEISS GeminiSEM 300), atomic force microscope (AFM, Oxford Instruments

Asylum Research Cypher ES) and transmission electron microscopy (TEM, FEI Talos F200X). In SEM test, the membrane samples were treated with spray-gold before the test and the surface were observed. As for AFM, the casting solution with a concentration of 1 wt% was prepared, and then an appropriate amount of this casting solution was dropped onto a mica sheet, which was subsequently vacuum-dried for 24 h at 60°C to obtain an AFM membrane sample. The membrane specimens were measured in a tapping mode to observe the microphase structure. Moreover, the microphase separation structures were characterized by transmission electron microscopy (TEM) with an accelerating voltage of 200 kV. Specifically, the membrane samples were stained by immersion in a 1 M Na_2WO_4 solution at room temperature for 24 h prior to testing. Then, the residual Na_2WO_4 was removed by washing 3 times with deionized water and dried at 60 °C overnight.

In addition, small-angle X-ray scattering measurements (SAXS, Austria Anton Paar SAXSess mc2) were conducted to further investigate the interdomain spacing of the AEMs. The average ion domain dimension formed by ion cluster aggregation was calculated according to the Bragg equation:

$$d = 2\pi/q \tag{2}$$

where q is defined as $(4\pi/\lambda)\sin(\theta)$ and 2θ is the scattering angle.

1.4 Ion exchange capacity (IEC)

The IEC was measured by the back-titration method. Before the test, a membrane sample in hydroxide form was vacuum dried at 60 °C for 24 h, subsequently soaked in a fresh standard solution of HCl (0.1 M) with a certain volume for 48 h to allow OH^- to convert into Cl^-. The HCl solution was then back titrated with a fresh standard solution of NaOH (0.1 M), using phenolphthalein as an indicator to determine the endpoint of the titration. The IEC (mmol g^{-1}) was calculated by the following Eq. (3):

$$\mathrm{IEC} = \frac{C_{\mathrm{HCl}}V_{\mathrm{HCl}} - C_{\mathrm{NaOH}}V_{\mathrm{NaOH}}}{m} \tag{3}$$

Where C_{HCl} and V_{HCl} are the concentration (mol L^{-1}) and volume (mL) of the HCl solutions, respectively. C_{NaOH} and V_{NaOH} represent the concentration (mol L^{-1}) and

volume (mL) of NaOH solutions, respectively. The m is the weight of the dried membrane sample.

1.5 Water uptake (WU), swelling ratio (SR) and hydration number (λ)

Water uptake (WU) and swelling ratio (SR) were determined by measuring the mass and dimension differences between fully hydrated and dried AEMs in the OH^- form. Briefly, the membrane samples were dried at 60 °C under vacuum for 48 h, and then the weight and dimension of the dried membrane samples were recorded. The dried membrane samples were soaked for 48 h in DI water at 25 °C, 40 °C, 60 °C and 80 °C. Then the membrane samples were taken out and quickly wiped the surface water, then the weight and dimension were quickly measured again. The weight of wet (m_{wet}) and dry (m_{dry}) membranes were used to evaluate the WU and was calculated according to Eq. (4):

$$WU(\%) = \frac{m_{wet} - m_{dry}}{m_{dry}} \times 100\% \qquad (4)$$

The swelling ratio (SR) of the membrane samples was evaluated based on the change between the wet membrane length (L_{wet}) and dry membrane length (L_{dry}), calculated by the following Eq. (5):

$$SR(\%) = \frac{L_{wet} - L_{dry}}{L_{dry}} \times 100\% \qquad (5)$$

The hydration number (λ) of the AEMs was calculated according to Eq. (6) based on WU and IEC.

$$\lambda = \frac{1000 \times WU}{18 \times IEC} \qquad (6)$$

1.6 Ionic conductivity (σ)

The hydroxide conductivity (σ) of the OH^- form AEMs was measured by an electrochemical workstation (PARSTAT 3000A, USA). The cut membrane samples were installed in a self-made battery module with two copper electrodes and fully immersed in boiling and degassed DI water that had been cooled to the temperature under test, reducing the influence of gas on the test results. The resistance R (Ω) of the sample membrane was measured by AC impedance method, with a test frequency of 0.1-10^5 Hz and test temperatures of 25°C, 40°C, 60°C, and 80°C. The OH^- conductivity of the samples was calculated according to Eq. (7):

$$\sigma = \frac{l}{R \bullet A} \quad (7)$$

Where l (cm) represent the space between the electrodes, A (cm^2) is the effective area of membrane.

The Arrhenius activation energy (E_a, kJ mol^{-1}) was calculated by the following Eq. (8):

$$E_a = -b \times R \quad (8)$$

where b is the Arrhenius curve slope as a function of the temperature and R is the ideal gas constant.

1.7 Mechanical properties and thermal stability

The mechanical properties of the AEMs were measured by a universal material testing machine (MTSCMT 8535) with a stretching rate of 5 mm min^{-1} at room temperature. Each membrane sample was repeated tested three times under the same conditions. A thermogravimetric analyzer (NETZSCH STA 449F5) was used to evaluate the thermal stability of the membranes. The membranes were heated under a nitrogen flow at the temperature ranging from 30-700 °C (10 °C min^{-1}).

1.8 Alkaline and oxidative stability

The alkali resistance stability of AEMs in OH^- form was conducted by immersing in 1 M NaOH solution at 80 °C for 1008 h. Upon completion of testing, the sample was taken out and washed thoroughly with DI water. The alkaline durability of the membrane was evaluated by the change of conductivity and the IEC remaining at 80 °C. Moreover, the OH^- form membrane was dried in vacuo at 80 °C to obtain the dry membrane for FT-IR spectra measurement and thermal stability. The alkaline durability of the membrane was further evaluated by the FT-IR spectra measurement and thermal stability.

The oxidative stability of AEMs was determined by immersion in Fenton's solution (4 ppm Fe^{2+} in 3% H_2O_2) to monitor the residual weight and conductivity. The sample membranes were soaked in the Fenton's solution for 8 h at 80 °C. After that, it was washed with DI water for 3 times to remove the residual Fenton's solution

on the membrane surface. Before measurement, the sample AEMs were immersed in 1 M NaOH solution for 48 h at room temperature and then dried under vacuum for 24 h at 60 °C.

1.9 Single cell performance

The single fuel cell performance of AEMs was tested by the fuel cell test system (Scribner 850e). The membrane electrode assemblies (MEAs) were prepared by catalyst-coated membrane (CCM) technology, as reported in detail in our previous work[34]. The catalyst ink was prepared using Pt/C (40 wt % Pt, Johnson Matthey) as catalyst and aPNB-TMA-1.68 (synthesized in our lab, Fig. S7) as the ionomeric binder for both cathode and anode. The catalyst and aPNB-TMA-1.68 were mixed in water-isopropanol solution followed by sonication for 2 h to obtain the catalyst ink dispersion. The mass ratio of catalyst to ionomer was 4 to 1. The catalyst ink was sprayed on the active area on both sides of the AEMs by an airbrush (Anest Iwata HPCH). The CCM with a Pt loading amount of 0.5 mg cm^{-2} was specifically sandwiched between two pieces of carbon paper (Toray TGP-H-060, Japan) to fabricate the MEA, which had an effective size of 2 cm × 2 cm. Finally, the MEA was installed into a single-cell module, whereupon the single fuel cell was operated at a temperature of 80°C with a fully humidified H_2/O_2 gas flow rate of 400/400 mL min^{-1}.

2. Experimental section

2.1 Synthesis of NB-O-Br monomer

The synthesis of NB-O-Br monomer was improved based on our previous work[34] (Scheme S1a).

2.2 Synthesis of NB-O-Hex monomer

The synthesis of NB-O-Hex monomer followed our previous work[14] (Scheme S1b).

2.3 Synthesis of allyl palladium chloride complex catalyst ((η^3-allyl)Pd(Cl)PPh_3)

The synthesis of (η^3-allyl)Pd(Cl)PPh_3 catalyst followed the approach outlined in our previous work[14] (Scheme S1c).

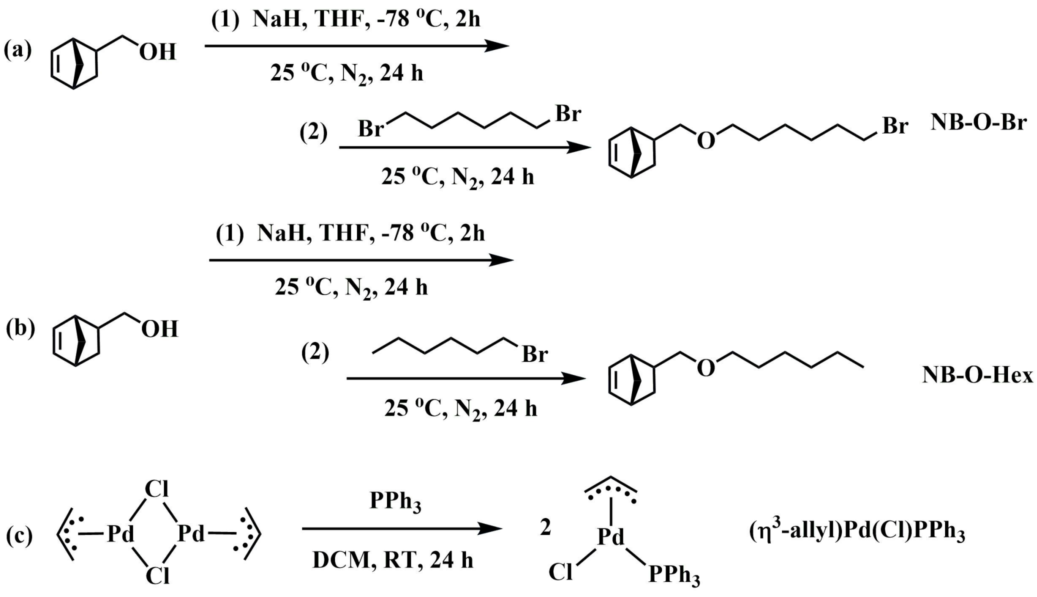

Scheme S1 Synthesis of (a) NB-O-Br monomer, (b) NB-O-Hex monomer, and (c) (η^3-allyl)Pd(Cl)PPh$_3$ catalyst.

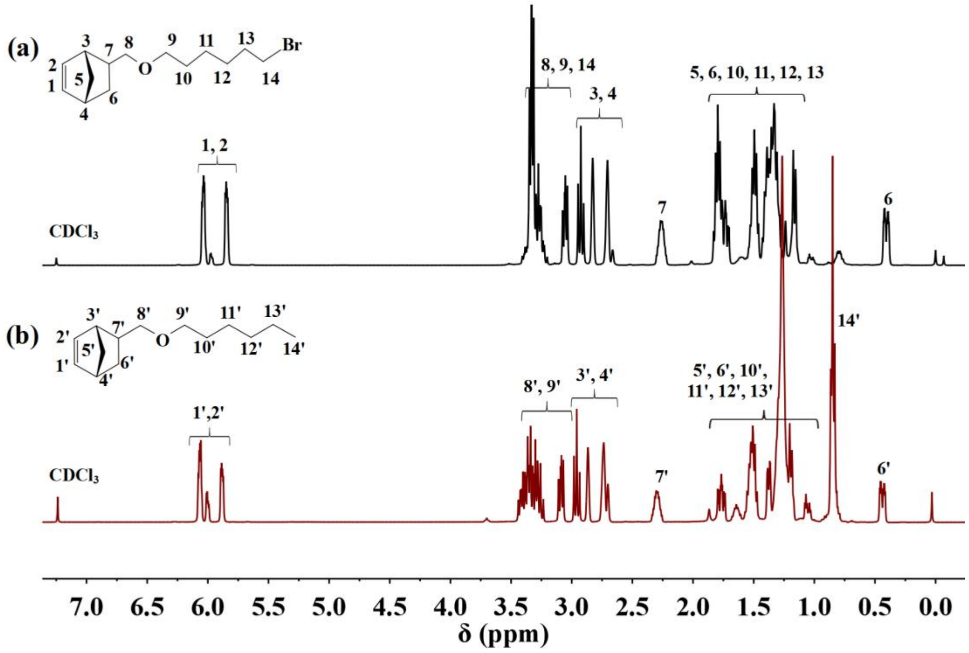

Fig. S1 ^{1}H NMR spectra of (a) NB-O-Br and (b) NB-O-Hex monomer.

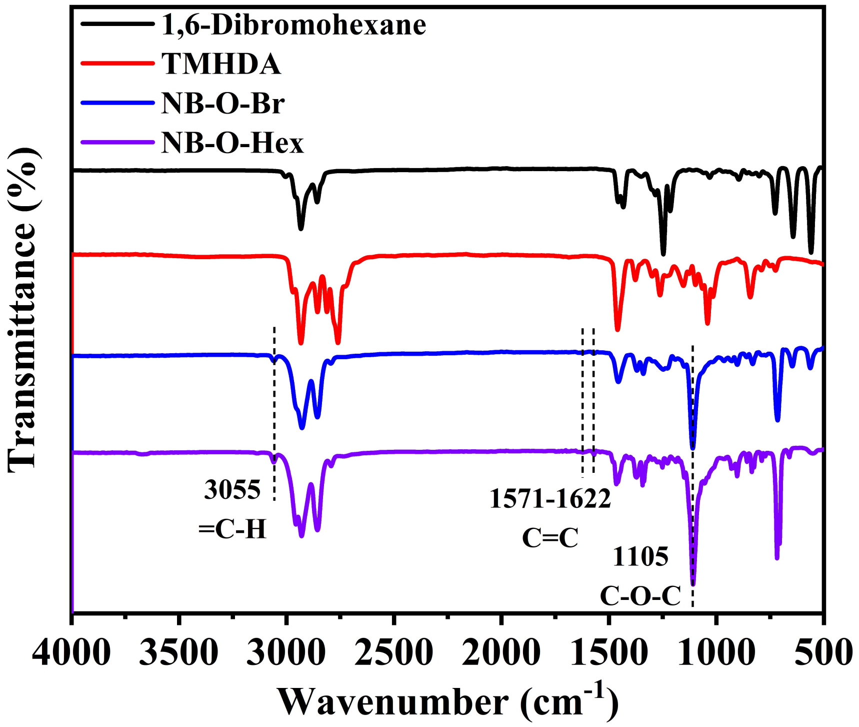

Fig. S2 FTIR spectra of 1,6-Dibromohexane, TMHDA, NB-O-Br and NB-O-Hex.

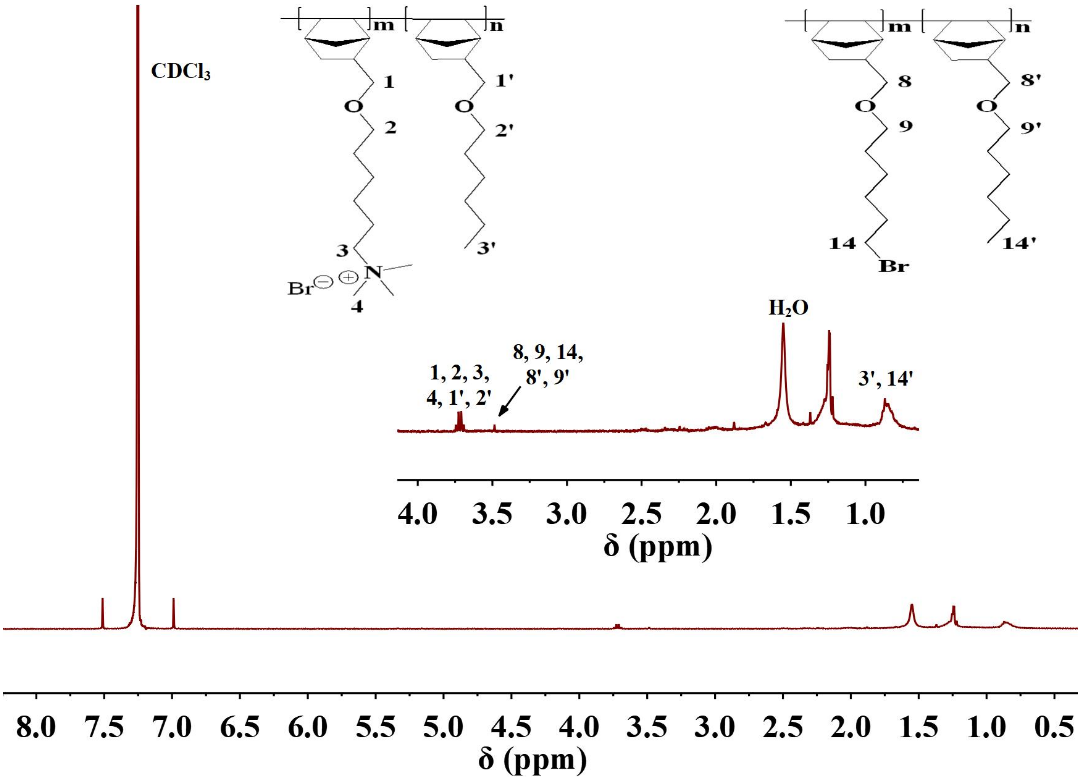

Fig. S3 ^{1}H NMR spectra of the residues in the solution after the GF test of AEM.

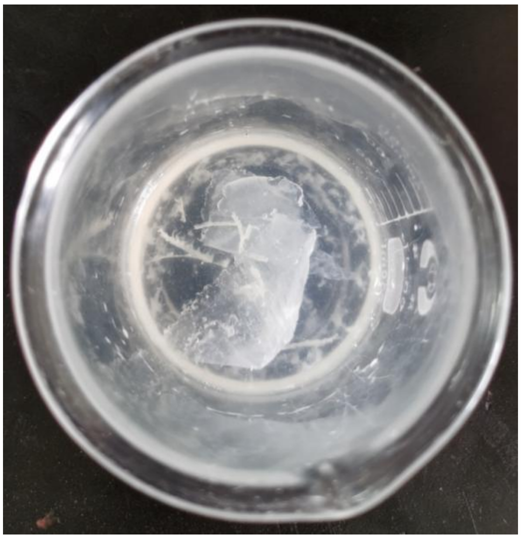

Fig. S4 The photograph of the swelling and rupture of the non-crosslinked membrane observed during the swelling ratio test at 40 °C.

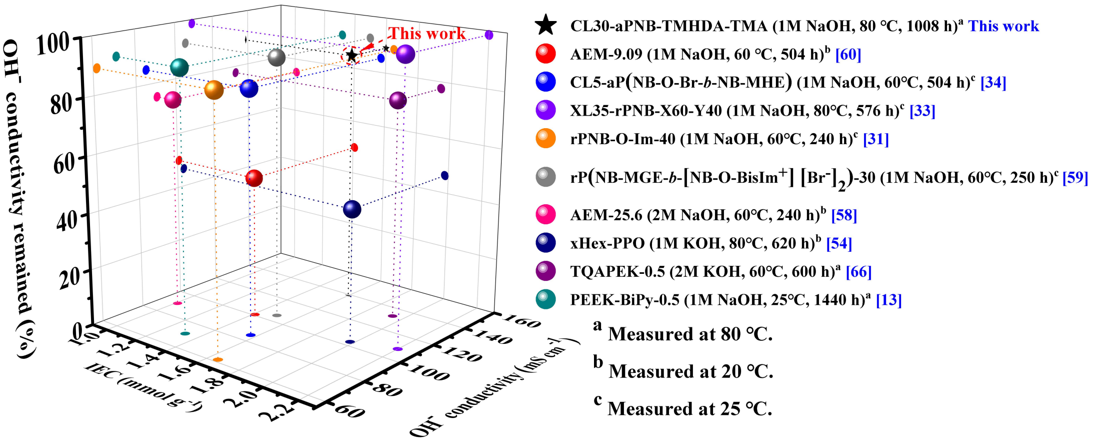

Fig. S5 Alkaline stability and conductivity of the membranes in this work and the literature[13, 14, 31, 33, 34, 54, 58-60, 66].

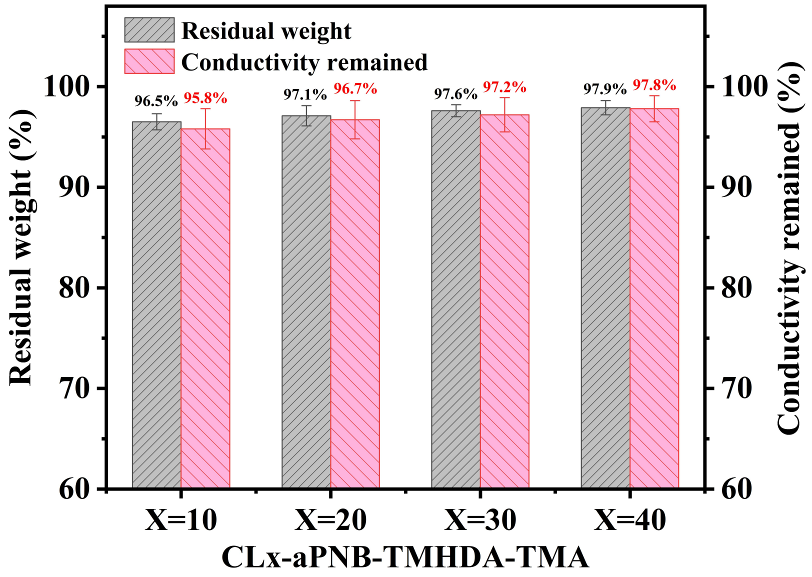

Fig. S6 Oxidative stability of the prepared AEMs immersed in Fenton's solution (4 ppm Fe^{2+} in 3 wt% H_2O_2) at 80 °C (remaining weight and conductivity).

2.4 Synthesis of aPNB-TMA-1.68 ionomer

The diblock copolymer aP(NB-O-Br-*b*-NB-O-Hex) (0.4 g) was completely dissolved in 10 mL THF solvent at room temperature. Subsequently, 1 mL trimethylamine solution (2 mol L^{-1} in THF) was added to the solution. The resulting homogeneous reaction mixture was then allowed to react at room temperature for 24 h under a N_2 atmosphere. After the reaction was completed, it was precipitated in excess n-hexane (200 mL), purified by further washing with DI water and dried at 60 °C under vacuum for 24 h. Before use, the ionomer was immersed in a 1M NaOH solution for 48 h at 25 °C to allow the halogen ions in the ionic polymer to be completely replaced by OH^-. Finally, the ionomer was denoted as aPNB-TMA-1.68 (IEC=1.68 mmol g^{-1}). And the chemical structures were characterized by the ^1H NMR spectrum (Fig. S7). Compared with aP(NB-O-Br-*b*-NB-O-Hex) copolymer, the signals at 2.94-3.59 ppm are attributed to the methyl and the methylene groups connected to the ether bond and quaternary ammonium functional group (H^1,H^2,H^3,H^4,$H^{1'}$,$H^{2'}$), and the peak at 0.83 ppm is associated with the methyl group at the end of the ether chain ($H^{3'}$). The positions and shapes of these characteristic peaks both confirmed that the quaternary ammonium cations were successfully grafted onto

the side chains of aPNB-TMA-1.68 ionomer.

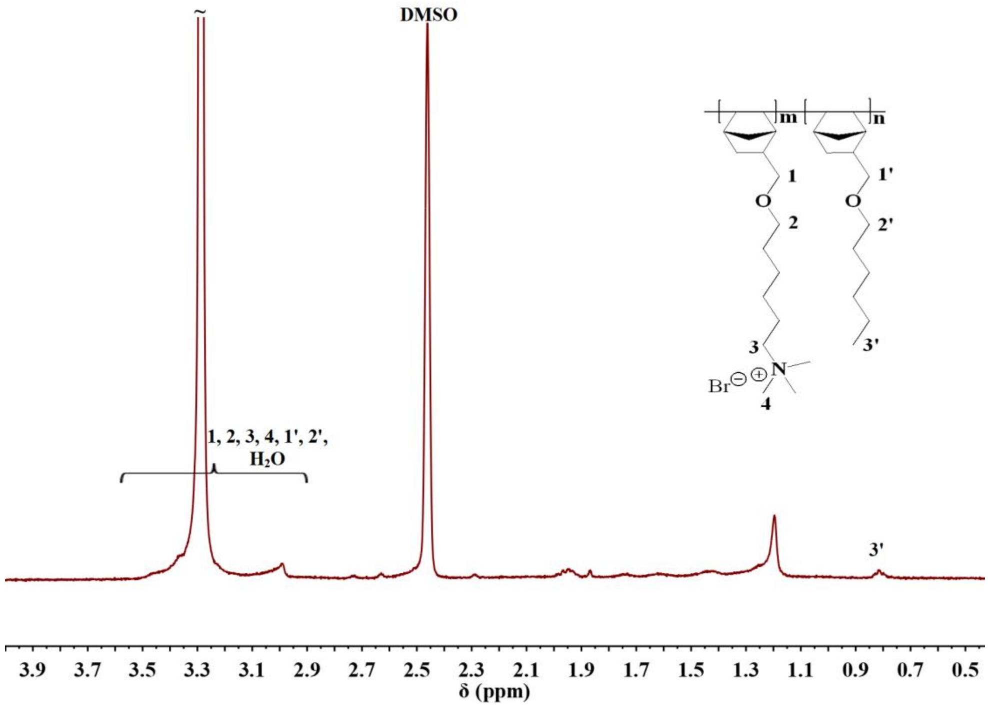

Fig. S7 ^{1}H NMR spectra of aPNB-TMA-1.68 ionomer.

References

[13] Y.J. Xu, C.H. Zhao, S.M. Huang, Y.L. Gan, L. Xiong, J.P. Zhou, H.B. Liang, Bis-pyridinium crosslinked poly(ether ether ketone) anion exchange membranes with enhancement of hydroxide conductivity and alkaline stability, Int. J. Hydrog. Energy 47(9) (2022) 6097-6110. https://doi.org/10.1016/j.ijhydene.2021.11.209.

[14] Q. Li, X.H. He, L. Huang, Y. Lu, S.Y. Zou, J. Ye, L.M. Huang, N.Q. Yu, Z.H. Fu, X.J. Zang, D.F. Chen, Porous PTFE supported bis(siloxane imidazole) functionalized Norbornyl copolymer composite anion exchange membrane for alkaline fuel cells, J. Appl. Polym. Sci. 140(40) (2023) 17. https://doi.org/10.1002/app.54495.

[31] S.M. Huang, X.H. He, C.W. Cheng, F. Zhang, Y. Guo, D.F. Chen, Facile self-crosslinking to improve mechanical and durability of polynorbornene for alkaline anion exchange membranes, Int. J. Hydrog. Energy 45(23) (2020) 13068-13079. https://doi.org/10.1016/j.ijhydene.2020.03.013.

[33] W.T. Chen, M. Mandal, G. Huang, X.M. Wu, G.H. He, P.A. Kohl, Highly Conducting Anion-Exchange Membranes Based on Cross-Linked Poly(norbornene): Ring Opening Metathesis Polymerization, ACS Appl. Energ. Mater. 2(4) (2019) 2458-2468. https://doi.org/10.1021/acsaem.8b02052.

[34] X.H. He, J.H. Zou, Y.F. Wen, B. Wu, X.J. Zang, J.H. Deng, Z.W. Qin, G.X. Yang, J. Xu, D.F. Chen, Preparation and performance of bisimidazole cationic crosslinked addition-type polynorbornene-based anion exchange membrane, Int. J. Hydrog. Energy 47(1) (2022) 69-80. https://doi.org/10.1016/j.ijhydene.2021.08.237.

[54] S. Sung, T.S. Mayadevi, K. Min, J. Lee, J.E. Chae, T.H. Kim, Crosslinked PPO-based anion exchange membranes: The effect of crystallinity versus

hydrophilicity by oxygen-containing crosslinker chain length, J. Membr. Sci. 619 (2021) 13. https://doi.org/10.1016/j.memsci.2020.118774.
[58] X.H. He, C.W. Cheng, S.M. Huang, F. Zhang, Y.P. Duan, C.Y. Zhu, Y. Guo, K. Wang, D.F. Chen, Alkaline anion exchange membranes with imidazolium-terminated flexible side-chain cross-linked topological structure based on ROMP-type norbornene copolymers, Polymer 195 (2020) 9. https://doi.org/10.1016/j.polymer.2020.122412.
[59] F. Zhang, X.H. He, C.W. Cheng, S.M. Huang, Y.P. Duan, C.Y. Zhu, Y. Guo, K. Wang, D.F. Chen, Bis-imidazolium functionalized self-crosslinking block polynorbornene anion exchange membrane, Int. J. Hydrog. Energy 45(23) (2020) 13090-13100. https://doi.org/10.1016/j.ijhydene.2020.03.046.
[60] C.W. Cheng, X.H. He, S.M. Huang, F. Zhang, Y. Guo, Y.F. Wen, B. Wu, D.F. Chen, Novel self-cross-linked multi-imidazolium cations long flexible side chains triblock copolymer anion exchange membrane based on ROMP-type polybenzonorbornadiene, Int. J. Hydrog. Energy 45(38) (2020) 19676-19690. https://doi.org/10.1016/j.ijhydene.2020.04.276.
[66] L. Li, C.X. Lin, X.Q. Wang, Q. Yang, Q.G. Zhang, A.M. Zhu, Q.L. Liu, Highly conductive anion exchange membranes with long flexible multication spacer, J. Membr. Sci. 553 (2018) 209-217. https://doi.org/10.1016/j.memsci.2018.02.048.
